# Parental adjustment to a burn-injured child: how to support their needs in the aftermath of the injury

**DOI:** 10.3389/fpsyg.2024.1456671

**Published:** 2024-09-30

**Authors:** Elisabete Cioga, Dulce Cruz, Carlos Laranjeira

**Affiliations:** ^1^School of Health Sciences, Polytechnic of Leiria, Leiria, Portugal; ^2^Centre for Innovative Care and Health Technology (ciTechCare), Polytechnic of Leiria, Leiria, Portugal; ^3^Comprehensive Health Research Centre (CHRC), University of Évora, Évora, Portugal; ^4^Nursing Department, University of Évora, Évora, Portugal

**Keywords:** burns, trauma, childhood, parenting, support, resilience, family-centered care, parental adjustment

## Abstract

The physical pain of a burn is immeasurable, but the pain of seeing a child suffer is indescribable. Childhood burns not only affect the child directly, but also have a significant impact on the parents and the rest of the family. During the acute phase of the burn, the child’s main allies in recovery are their parents, so they face emotional turbulence, having to support their children while witnessing the painful procedures they go through. They often feel helpless and distressed because they are unable to protect their children from suffering and have difficulty managing everything they feel. In addition to the often-present feeling of guilt, they also experience sadness and worry, particularly when returning home. After hospital discharge, fears increase, as do the challenges. There is a need to readapt the entire family dynamic to respond to the needs of the burned child who returns home. This readaptation often generates stress and anxiety, interfering with the entire family structure. It is crucial to try to understand these parents and give them all the support they need. Only capable and well-adjusted parents can ensure resilient family environments with safer and calmer children, thus promoting family well-being. In this perspective paper, the authors underline the role of parents of burnt children during the long trajectory of child rehabilitation and recovery. By acknowledging their needs, feelings and challenges healthcare providers can engage and support suffering parents toward more family-centered approach.

## 1 Introduction

Burns are the fourth most common type of trauma in the world and a leading cause of mortality and disability in developing countries (Sasor and Chung, 2019). According to the World Health Organization (WHO), burns cause more than 180,000 deaths per year, mainly in developing countries (Smolle et al., 2017). In Europe, 50–80% of all burns in children affect children under the age of five (Kashega et al., 2016; Lernevall et al., 2021). Hot fluids and surfaces, chemicals, flames, and electrical sources are the primary factors that commonly lead to burn injuries (Jeschke et al., 2020). Children are especially vulnerable due to their age, and burns are the fifth most prevalent source of non-fatal injuries in childhood. They are at an early stage in their life cycle where they are naturally curious and seek to explore the world around them. This makes them more likely to get involved in situations that can result in trauma, especially accidental burns and when not supervised by an adult. At the same time, their immaturity and lack of understanding of danger make them more susceptible to risky situations without realizing the consequences. The injuries are influenced by a range of factors, including individual actions, family circumstances, and wider social and regulatory components (Nassar et al., 2023; Oliveira et al., 2015). For example, situations in households, particularly those that include cooking or open fires, have consistently been identified as significant areas of risk. Furthermore, specific cultural customs, insufficient public knowledge, and restricted availability of safety gear exacerbate the dangers (Nassar et al., 2023).

Although childhood burns are preventable, they impact everyone involved, and it is important to educate parents and caregivers about preventive measures to create safe environments (Kashega et al., 2016). A burn is one of the most painful and traumatic injuries a child can suffer, and is also a very distressing event for parents, with great impact on the entire family (Al-Naimat and Abdel Razeq, 2022; Sveen et al., 2017; Wickens et al., 2024). Burns are an important predictor of risk in the development of children. Depending on their severity and extent, burns can result in serious sequelae and important functional, aesthetic, psychological and social limitations, in addition to a high mortality rate (Barbieri et al., 2016; Oliveira et al., 2015). Burn injuries bring about a multiplicity of stressors with physical and biopsychosocial impact, compromising individual and family dimensions.

In fact, parents have a crucial role as primary sources of support during the acute phase (Simons et al., 2019), and throughout the process from the occurrence of the accident to the return home. These children require prolonged hospitalizations, which affects the quality of family life and changes their daily routine (Al-Naimat and Abdel Razeq, 2022; Oliveira et al., 2015), leading to an inevitable break with previous family life (Bakker et al., 2013).

## 2 What do these parents feel?

Burns have a significant physical effect but also a profound influence on the psychological and emotional well-being of the child and their family, being experienced as a traumatic event (Lernevall et al., 2021; Lernevall et al., 2020; Patel et al., 2022). Studies show that burns cause psychological effects on parents (Bakker et al., 2013; Oliveira et al., 2015), with indirect impacts on the child’s health (De Young et al., 2014). Good family functioning is therefore important to improve outcomes for children who have suffered a burn (Sveen et al., 2017). Burn injuries sustained by children can have a notable psychosocial effect on parents, particularly mothers, impacting their physical health and psychological welfare (Heath et al., 2018). Parents may undergo reactive distress, which can manifest as anxiety, anger, depression, guilt, marital and financial difficulties, post-traumatic stress symptoms (PTSS), and an increased burden of responsibility (Bakker et al., 2013; Bayuo and Wong, 2021; Lernevall et al., 2021; Oliveira et al., 2015; Öster et al., 2014; Sveen et al., 2017; Wickens et al., 2024; Willebrand and Sveen, 2016). This is often due to the need to divide their time between caring for undamaged siblings, work, hospital visits, and managing household tasks. Symptoms of acute stress are also common during the first months after the accident and can last up to 2 years in fathers and up to 10 years in mothers (Öster et al., 2014).

Studies indicate that how parents handle difficult situations is a strong indicator of how well their children will adapt to their injuries (Foster et al., 2020). Support of family members can influence the child’s adherence to medical treatment and, therefore, their psychological and physical healing (Santos et al., 2024). Positive social ties are associated with improved adaptation; however, excessive social support can have a detrimental impact, as patients may become too dependent on caretakers and lose motivation. Consequently, if the unwounded experience challenges in dealing with and comprehending the trauma, this can hinder the recovery and adaptation of those who were damaged (Wisely and Gaskell, 2012). Therefore, parents play a crucial and essential role in providing medical treatment for their children and supporting their psychological and social rehabilitation after a burn injury.

Living with the child’s suffering and regularly carrying out invasive therapeutic procedures leads to great suffering, making parents feel their children’s pain (Oliveira et al., 2015). When they see their children undergoing painful procedures, they need to deal with their own feelings and fears, and at the same time support their children (Foster et al., 2020; Lernevall et al., 2020). All of this makes parenting a great challenge and generates great anxiety, which often translates into a reduced ability to care for their children and effectively satisfy their perceived needs (Foster et al., 2020). For parents, experiencing and/or being involved in their children’s medical procedures results in a traumatic experience, a fact made worse by the overload due to other existing responsibilities, such as caring for other children, other family members, professional commitments, among others (Lernevall et al., 2023).

Mothers often express tiredness and overload of responsibilities due to the many difficulties and pressures placed on the family. For some, this persists years after the burn, due to the multiple medical consultations and treatments (Wickens et al., 2024). Mothers, in particular, are often blamed for the accident, further exacerbating their feelings of helplessness and anguish (Lernevall et al., 2021). Seeing your child in a hospital bed, wrapped in bandages, needing long periods of fasting for surgical debridement procedures, and unable to run or play like a healthy child, undermines the feeling of competence and parental care, often awakening feelings of guilt (Oliveira et al., 2015). Similarly, parents reported feelings of loneliness, social isolation, helplessness, hopelessness, and almost abandonment in the long journey of caring for a burned child (Al-Naimat and Abdel Razeq, 2022; Bayuo and Wong, 2021; Egberts et al., 2019; Kashega et al., 2016; Lernevall et al., 2023; Oliveira et al., 2015; Öster et al., 2014).

Children who are burn victims depend especially on their parents to meet their physical, emotional, and social needs (Foster et al., 2020), therefore, parents are a fundamental element in their rehabilitation and adaptation (Oliveira et al., 2015). Parents play a unique and absolute role in their children’s medical care and psychosocial recovery, especially in emotional management (Heath et al., 2019), however, they report and feel a decrease in the ability to meet their children’s needs, in maintaining their role as caregiver, so that the physical and psychological adaptation of the sick child and the well-being of the entire family unit may be threatened (Foster et al., 2017).

Overall, qualitative evidence supports three distinct phases that describe the trajectory of parents of burn-injured children: (1) experiencing the burn event, (2) inpatient care, and (3) post-discharge care (Ravindran et al., 2013a, 2013b; McGarry et al., 2015; Santos et al., 2024). In the first phase, parents describe the child’s burn event as highly distressing, although they repressed their emotional reactions until their child received medical care. The inpatient phase poses the greatest difficulties for parents, especially the observation of invasive and traumatic medical interventions. These procedures cause considerable parental anguish and are recognized as the most distressing part of the entire experience. In the last phase, when children return to the community, the sense of comfort that comes with starting regular routines can be eclipsed by parents’ sense of responsibility with providing optimal after-care for their child. Furthermore, parents report concerns regarding the wound healing process (fear of permanent scarring) and express reduced trust in their parenting skills. Thus, discharge planning is a crucial component, because parents feel isolated during this period and believe they lack sufficient knowledge about their child’s condition (Santos et al., 2024). This approach highlights a family-centered care that prioritizes the healthcare of the whole family over individual care (Bayuo and Agbeko, 2023).

## 3 Family dynamics, what changes?

As previously mentioned, burns affect not only the child, but also the entire family. The importance of family dynamics in the recovery of children with burns has been duly documented, noting that relationships between different family members can positively or negatively influence both the physical and emotional recovery of the child (Öster et al., 2014; Parrish et al., 2019). Parents’ routines and schedules require permanent adaptation, and family dynamics are changed. Parents of burned children experience profound changes in their parental roles, family relationships and ability to care for their other children (Al-Naimat and Abdel Razeq, 2022). Parents’ concern and focus on the hospitalized child affects their ability to support other children in the family (Bayuo and Wong, 2021).

Often, during the hospitalization period, the siblings of children who suffered the burn stay with other family members. When the burned child returns home, the siblings feel neglected by their parents, as these spend many hours caring for their sick sibling, which is why some negative reactions have been documented (Öster et al., 2014). Siblings simultaneously expressed jealousy and feelings of being outsiders, leading to disruptive emotional responses (Phillips et al., 2007; Bayuo and Wong, 2021). Siblings of childhood burn patients are labelled “forgotten children” because they experience significant psychosocial distress and are isolated from support systems inside and outside the family (Joosten et al., 2019). However, evidence also suggests that siblings of child burn survivors may become more resilient in the process of normalizing everyday life and adjusting to new life circumstances (Öster et al., 2014).

Accompanying a hospitalized child often forces parents to be physically apart from their remaining family, leading to moments of ambiguity, as they need to accompany their burned child and, at the same time, miss and worry about their spouse and the other children who remained at home (Oliveira et al., 2015).

These feelings lead parents to look for ways to restore family well-being and try to have a normal life, which requires adaptation from the entire family. The relationship between parents can be affected: some they become closer but other times end up getting divorced, with the burn event intensifying pre-existing problems (Lernevall et al., 2020).

As a form of adaptation, parents report changing their perspectives about anxiety-producing situations and how they experience their child’s injury. They changed their expectations regarding the injured child, tried different parental approaches, and faced difficulties in establishing limits (Barbieri et al., 2016; Foster et al., 2019, 2020). The literature also explains how changes in parental behaviors are visible through excessively overprotective attitudes and transformations in the family structure to accommodate the child (Barbieri et al., 2016). Some mothers adopt protective behaviors towards their children, which in turn can promote avoidant survival strategies (Wickens et al., 2024). “Adjustments to parenting impact family function and dynamics, and while some adaptations may promote family cohesion and flexibility after the burn, others may contribute to stress, for example, by promoting discord between siblings due to different parental treatment” (Foster et al., 2020, p. 1208).

Upon hospital discharge, research indicates a high level of uncertainty over the parent’s capacity to provide for the injured child. While being discharged from the burn unit was seen as a significant achievement, Bäckström et al. (2018) observed that, after leaving the hospital, family care providers encountered a lack of organized assistance. They experienced a sense of isolation and neglect, coupled with a lack of confidence in outpatient programs. Following discharge, the subsequent phase was characterized by chaos, feelings of lowered self-worth, powerlessness, tension, and dejection, as well as the challenges of coping with helplessness and a loss of hope (Ravindran et al., 2013a). After being released from the hospital, the parents expressed anxiety and concern for their child’s future, including their ability to get married and become independent as they mature (Ravindran et al., 2013a). As injuries were healed and formed scars, parents had to protect their children from negative judgments, prolonged stares, and mockery from society (Bayuo and Wong, 2021; Rimmer et al., 2015).

## 4 But what do parents of burn-children need?

Parents have multiple needs and concerns during the long process of treatment and recovery, so all support will be essential for both the parents’ well-being and the child’s speedy recovery (Lernevall et al., 2023; Pan et al., 2015). Psychological support is recognized as important for all family members (Wickens et al., 2024). Family-centered care is a tool where the family is considered a partner throughout the process and that improves collaboration between all parties involved. Family members negotiate their level of participation and how they wish to provide care. On the other hand, helping the family to identify close friends they can turn to increases the possibility of obtaining more support, both practical and psychological (Al-Naimat and Abdel Razeq, 2022; Wickens et al., 2024). Lernevall et al. (2023) identified four support needs of parents of children with burns: (a) caring for the family as a whole; (b) feeling calm and safe during care provided by health professionals; (c) making time for one’s own needs; and, finally, (d) feeling some control over the situation (Lernevall et al., 2023). In this context, it is important to prepare families for trauma recovery by identifying healthy coping mechanisms and facilitating the expression of emotions after a distressing event (Lernevall et al., 2023; Wickens et al., 2024). Table 1 presents some parental strategies to promote resilience and adjustment in children who are burn survivors.

**Table 1:** Parenting strategies to promote resilience and adjustment in child burn survivors (Foster et al., 2019; Van Niekerk et al., 2020).

Engaging in interactive and enjoyable experiences with child burn survivors, such as play, conversation, entertaining activities, and joint endeavors. Ensuring that the child experiences affection, security, and appreciation.
Fostering the child burn survivor’s self-esteem and confidence by granting them an appropriate level of autonomy and independence. Motivating the child to exercise autonomy following the burn incident, even if the choices they make are of minor significance. Motivate the child to employ problem-solving skills when confronted with challenges. Commending the child when they strive to effectively tackle obstacles.
Reacting to difficulties in a constructive manner. Verbalizing thoughts can be advantageous in aiding the child’s comprehension of cognitive processes. Embracing the fact that the burnt child’s appearance may have changed. Child may experience challenges in accepting their physical appearance as being distinct from that of their peers. Primarily, the burnt child must feel acceptance from the parents.
Overcoming challenges fosters personal development. Assist child in recognizing their individual skills and exploring new hobbies, fostering a sense of optimism for the future.
Collaborate with child burn survivor’s school to ensure that the staff is adequately prepared to provide support for the child upon their return to school.
Establishing connections with family, community, or religious networks for assistance. Ensuring that the child receives assistance and encouragement from immediate family members and cherished individuals. Consider facilitating social interactions between the child and other children who have experienced a burn damage.

Given the impact of parenting on family resilience and well-being, there is also a clear need for parental support during recovery to prevent potential adverse outcomes for family dynamics, especially when a burned child has permanent functional and behavioral limitations (Foster et al., 2020). Therefore, it is extremely important to screen parents’ psychological distress in order to develop supportive psychosocial interventions (Bayuo and Wong, 2021; Van Niekerk et al., 2020). In this vein, Heath et al. (2018) showed that peer support had a substantial influence on the psychosocial rehabilitation of parents of burn-injured children, offering them encouragement, inspiration, hope, and reassurance. Adhering to the notion of helper therapy and providing assistance to others can lead to a beneficial cycle of improvement for the helper. Additionally, those who receive support can also become helpers, thereby increasing the group’s available resources and strengthening social connections (Dukes et al., 2022). In the future, it is imperative to promote online assistance for parents of burn-injured children. Sharing personal experiences with peers has promising and exhilarating prospects for empowering parents. A resource of this nature might offer easily available and personalized guidance for supportive care, which can be informed and advocated by experts in the field.

Adopting a family centered care framework (Egberts et al., 2019; Wickens et al., 2024) and using the International Classification of Functioning, Disability and Health (ICF; World Health Organisation, 2001), many healthcare professionals (HCP) have shifted their approach from interventions that only target physical and emotional limitations of disabled children towards interventions that focus on performance and participation-focused practices (Anaby et al., 2021). Additionally, these HCP collaborate with parents in providing therapeutic interventions that is integrated into the child’s daily life. Family-centered parent participation programs (Chow et al., 2024) demonstrate significant potential in effectively addressing anxiety and preparing parents for the rehabilitation phase. Studies have shown that structured psycho-educative interventions, such as cognitive restructuring, seeking social support, and problem-solving, when combined with non-structured support groups, effectively target the psychological needs of families and provide them with the essential coping mechanisms to explore and treat potential catastrophizing interpretations of reality (Egberts et al., 2020). Furthermore, initiatives that are focused on systemic approaches, such as Burns Camps, show potential in terms of promoting recovery for the entire family (Armstrong-James et al., 2019). “Child–parent–relationship therapy (CPRT) is also an evidence-based approach for working with families that focuses on enhancing the parent–child attachment while lowering parental stress” (Rodríguez et al., 2021, p. 1) and reducing overall behavioral concerns for the child (Bratton and Landreth, 2019).

## 5 Final remarks

Childhood burns are not just a clinical challenge, they have a significant impact on the emotional and psychological well-being of everyone involved. Clearly, burns affect not only the child, but they also have profound implications for family dynamics. Parents face a mix of emotions, from guilt and anxiety to emotional exhaustion, as they try to offer support to their child and maintain stability in their family life. Changes in parental roles, family relationships and daily routine are evident and often challenging for all family members and caregivers.

Assuming that integrated family-centered care is considered of utmost importance in the context of pediatric burn injuries, its adoption should be assumed by healthcare professionals when reducing parental distress and addressing the children’s difficulties adjusting emotionally and psychosocially. Early intervention and ongoing care should be underlined, given the complex needs of these families and the persistence of parental distress over time. To ensure consistency in this approach, we recommend a multidisciplinary assessment using patient-reported outcome measures (PROMs) and other psychosocial screening instruments to improve the health and psychological outcomes of both parents and burn-injured children. Further empirical research is needed to ascertain the potential influence of a burn-specific family-centered model in the evaluation and intervention of pediatric patients and their families. This framework should consider individual perceptions/experiences and provide specific interventions for each stage of care (including peri-trauma, acute medical care, rehabilitation and discharge from care) to support and expedite child recovery and parental adjustment.

Moreover, specific attention should be given to how parents can support their child’s recovery, for example, by providing psychoeducation and practical support for parents throughout the treatment and recovery process. Care coordination and adequate support can make all the difference, not only helping parents cope with their own emotions and stress, but also facilitating the child’s recovery and promoting family cohesion. In this sense, specific intervention strategies aimed at supporting parents are essential to strengthen family resilience and improve long-term results. As such, there is a pressing need for a holistic approach to treating childhood burns. By recognizing and addressing the emotional and psychological impacts of burns on the family, we can truly promote the well-being of everyone involved and ensure a better quality of life for children and their families.

## Data Availability

The original contributions presented in the study are included in the article/supplementary material, further inquiries can be directed to the corresponding author.
